# Rituximab-induced HMGB1 release is associated with inhibition of STAT3 activity in human diffuse large B-cell lymphoma

**DOI:** 10.18632/oncotarget.4816

**Published:** 2015-07-31

**Authors:** Tiansuo Zhao, He Ren, Xiuchao Wang, Pengfei Liu, Fan Yan, Wenna Jiang, Yang Li, Jing Li, John G. Gribben, Li Jia, Jihui Hao

**Affiliations:** ^1^ Key Laboratory of Cancer Prevention and Therapy, Tianjin Medical University Cancer Institute and Hospital, National Clinical Research Center for Cancer, Tianjin, China; ^2^ Department of Lymphoma, Tianjin Medical University Cancer Institute and Hospital, National Clinical Research Center for Cancer, Tianjin, China; ^3^ Key Laboratory of Cancer Immunology and Biotherapy, Tianjin Medical University Cancer Institute and Hospital, National Clinical Research Center for Cancer, Tianjin, China; ^4^ Centre for Haemato-Oncology, Barts Cancer Institute, Queen Mary University of London, London, UK

**Keywords:** DLBCL, rituximab, immunogenic cell death, HMGB1, STAT3

## Abstract

Treatment with rituximab plus cyclophosphamide, doxorubicin, vincristine, and prednisone (R-CHOP) has greatly improved clinical outcomes in patients with diffuse large B-cell lymphoma (DLBCL) compared with CHOP. The mechanism of rituximab-induced cell death is poorly understood. We found that rituximab does not enhance the directly killing efficacy of CHOP, as tested on a panel of DLBCL cell lines. Rituximab induced a rapid release of HMGB1 (High mobility group protein B 1). This release is independent of cell death but significantly correlated with an inhibition on STAT3 activity. In the resting state, HMGB1 co-localizes and interacts with STAT3 in the nucleus of DLBCL cells. Treatment with rituximab breaks this binding and triggers HMGB1 release. Treatment with R-CHOP but not CHOP significantly increased plasma HMGB1 and decreased IL-10 concentrations in DLBCL patients compared with controls. The conditioned medium from rituximab-treated DLBCL cells is able to trigger dendritic cell maturation, phagocytosis, and IFN-g secretion by cytotoxic T cells. In conclusion, our results demonstrate that rituximab induces an inhibition on STAT3 activity, leading to increased HMGB1 release and decreased IL-10 secretion, which elicits immune responses, suggesting that indirect effects on the immune system rather than direct killing contribute to elimination of DLBCL.

## INTRODUCTION

Diffuse large B-cell lymphoma (DLBCL) and follicular lymphoma (FL) are the most common forms of aggressive and indolent non-Hodgkin lymphomas (NHLs), respectively. The first-line therapy for DLBCL and FL is rituximab, a monoclonal anti-CD20 antibody, plus cyclophosphamide, doxorubicin, vincristine, and prednisone (R-CHOP) [1, 2]. Treatment with R-CHOP achieved high response rates and led to significant improvements on overall survival rates in patients with NHLs compared with those treated with CHOP alone [3–6]. In some of B-cell malignancies, rituximab alone can induce high overall response rates and long term remissions [7, 8]. Despite its undeniable value as a component of therapy for anti-B cell malignancies, the mechanisms of action responsible for rituximab's anti-tumor effects are not fully understood [9].

Over the last decade, the cell killing modalities of anti-CD20 antibodies on malignant B-cells were considered as complement-dependent cytotoxicity, antibody-dependent cellular cytotoxicity, apoptosis, inhibition of STAT3 activity, and lysosome permeability-mediated cell death [3, 9–12]. However, *in vitro* studies showed that rituximab is the weakest killer on malignant B-cells among anti-CD20 antibodies [10, 13, 14]. The cell-killing modality of rituximab is still elusive. So far, there is little convincing evidence to show that the anti-tumor effect of rituximab is mediated by direct killing to malignant B-cells. Previous reports showed that the anti-CD20 antibody-treated lymphoma cells are taken up and processed by antigen presenting dendritic cells (DCs) with subsequent cross-presentation of tumor-derived antigens to T cells [15–17]. This suggests that anti-CD20 antibodies may have a ‘vaccinal effect’ and exert therapeutic effects through the induction of an adaptive cellular immune response. However, the precise mechanism by which the anti-CD20 antibody induces immune responses is also unclear.

In recent years a new concept ‘immunogenic cell death’ (ICD), a cell death modality that stimulates immune response against dead cell antigens, has drawn great attention in the field of anticancer therapy. The immunogenic characteristics of ICD are mainly mediated by damage-associated molecular patterns (DAMPs), which include pre-mortem surface exposed calreticulin (CRT), secreted ATP, and post-mortem released high mobility group protein B1 (HMGB1) after the exposure to certain cytotoxic agents. These danger signals are recognized by antigen-presenting cells such as DCs followed by the formation of T cell-mediated adaptive immunity [18–22].

HMGB1 is a non-histone chromatin protein and universally expressed by all nucleated cells. It can be actively secreted by cells of the innate immune system in response to pathogenic products and passively released by injured cells as they succumb to primary or secondary necrosis [23–25]. Extracellular HMGB1 has emerged as a key mediator in the regulation of immune responses to infection and sterile injury [26]. The release of HMGB1 by dying cancer cells is mandatory to license host DCs to process and present tumor antigens. Extracellular HMGB1 interacts with Toll-like receptors (TLRs) and receptor for advanced glycation end products (RAGE) on the DCs, which are involved selectively in the cross-priming of anti-tumor T lymphocytes *in vivo* [27, 28]. It has been reported that the type II anti-CD20 antibody GA101 induces both programmed cell death and HMGB1 release from Raji lymphoma cell line. The conditioned medium from GA101-treated cells elicits maturation of DCs [29]. However, Rituximab showed less cytotoxic effect on Raji cells.

On the basis that rituximab induces immune response *in vivo*, we hypothesized that treatment with rituximab might elicit ICD in malignant B-cells. To test this postulate, we compared cell death modalities between CHOP and R-CHOP-treated DLBCL cells and determined rituximab-induced HMGB1 release from DLBCL cells *in vitro* and *in vivo*. We found that rituximab-induced HMGB1 release from a panel of DLBCL cell lines is associated with inhibition of STAT3 activity.

## RESULTS

### Rituximab does not enhance CHOP-induced cell death in DLBCL cells

**Figure 1:**
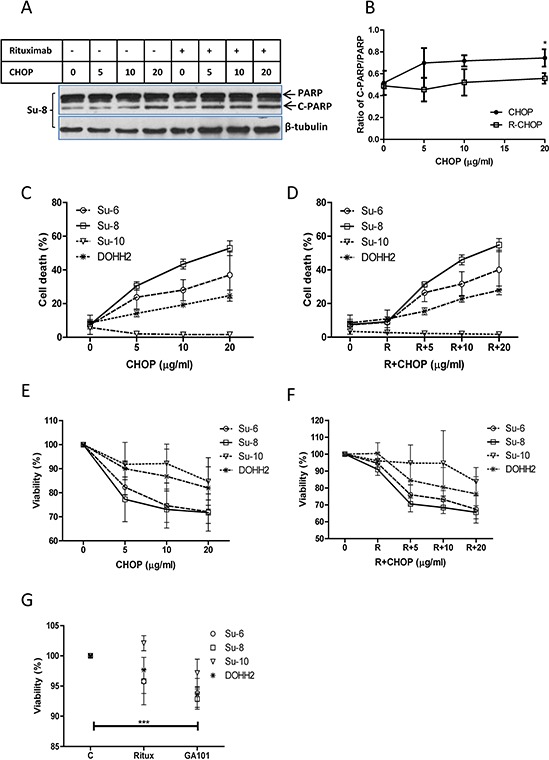
Comparison of CHOP and R-CHOP-induced killing in DLBCL cell lines DLBCL cell lines were treated with 5, 10, or 20 μg/ml of CHOP, 10 μg/ml of rituximab, or R-CHOP for 24 hours. **A.** PARP cleavage. A group of representative Western blots of PARP cleavage induced by CHOP or R-CHOP. ‘PARP’ means full length PARP (MW = 116) and ‘C-PARP’ indicates cleaved PARP (MW = 86). β-tubulin was used as a loading control. **B.** Statistical analysis of PARP cleavage. Ratios of cleaved PARP to PARP were analyzed by densitometry. Data shown were mean ± SD from 4 different cell lines. ‘*’ means significantly increased PARP cleavage in 20 μg/ml CHOP-treated groups compared with their controls. **C** and **D.** CHOP (C) or R-CHOP (D) induced cell death. Cells were stained with 7-AAD and 7-AAD positive cells were determined by flow cytometry as dead cells. **E** and **F.** CHOP (E) or R-CHOP (F) –mediated cytotoxicity. After treatment with CHOP or R-CHOP for 48 hours, decreased viability (cytotoxicity) was determined by CCK-8 assay. **G.** Rituximab or GA-101-induced cytotoxicity. Cells were treated with 10 μg/ml rituximab (Ritux) or GA-101 for 48 hours and the cytotoxicity was determined by CCK-8 assay. Significantly increased cytotoxicity in GA-101-treated group was analyzed using means from 4 different cell lines. (C–F) Data shown were mean ± SD from 3 independent experiments.

Firstly, we compared the killing efficacies of R-CHOP and CHOP on malignant B-cells *in vitro*. Four DLBCL cell lines, Su-6, Su-8, Su-10, and DoHH2, were treated with 3 different doses of CHOP and/or 10 μg/ml of rituximab for 24 hours. Treatment with 20 μg/ml of CHOP induced a significantly increased PARP cleavage, a marker for apoptosis. However, there was no significant difference in the amount of PARP cleavage between CHOP and R-CHOP-treated cells (Figure 1A and 1B, Supplemental Figure 1A). The four cell lines showed differential sensitivities to CHOP or R-CHOP-induced cell death and cytotoxicity (Figure 1C–1F), but again there was no statistical difference between the treatment with CHOP and R-CHOP, as analyzed by ANOVA (*P* > 0.05). GA-101, another anti-CD20 antibody, significantly induced cytotoxicity on DLBCL cells but rituximab failed to do so (Figure 1G). These results demonstrate that rituximab may not kill DLBCL cells directly.

### Treatment with rituximab induces a rapid HMGB1 release from DLBCL cells

**Figure 2:**
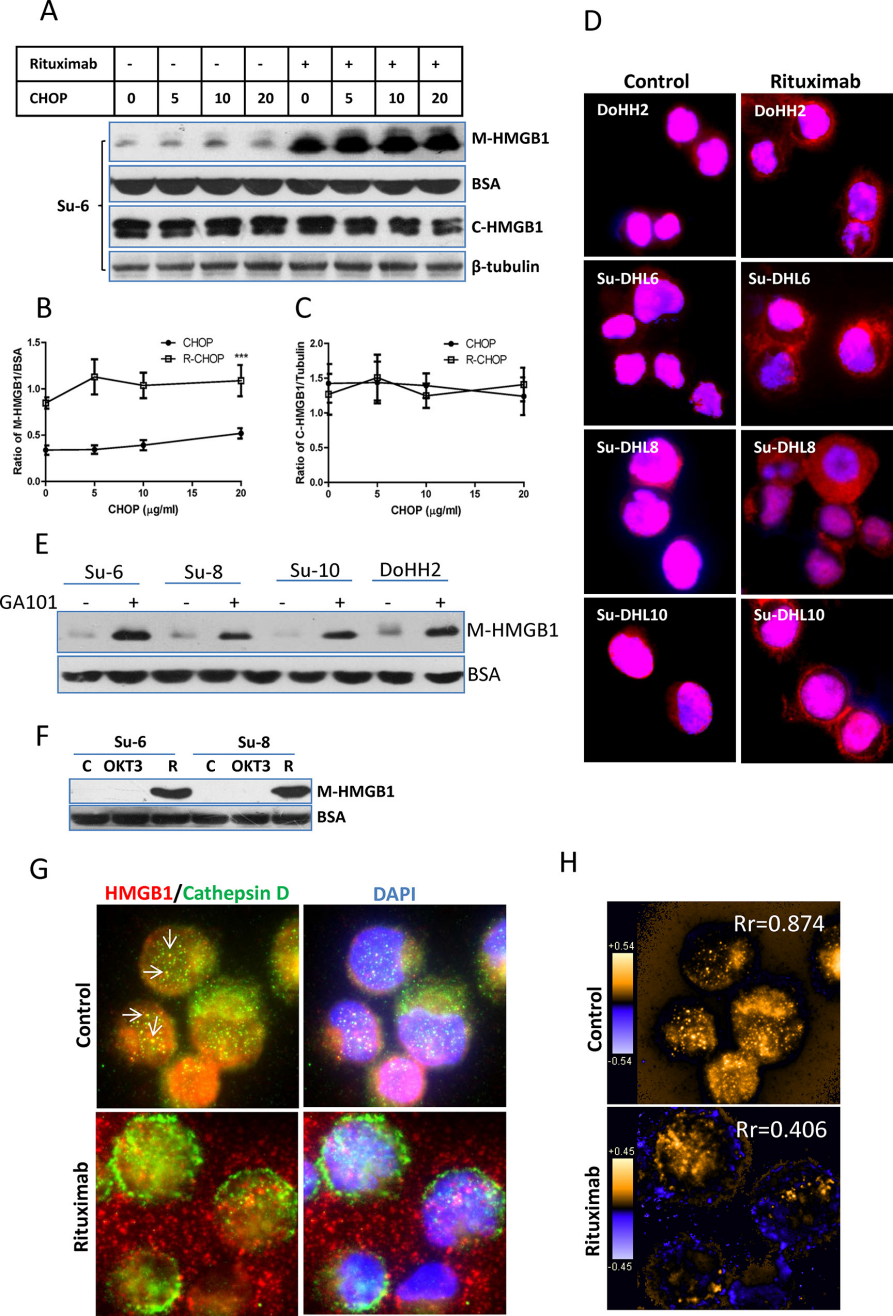
Rituximab induced HMGB1 release **A-C.** Four DLBCL cell lines in 2 × 10^6^/ml were treated with rituximab for 4 hours. HMGB1 in the conditioned medium (50 μl) and cytosolic extracts were determined by Western blotting using a mouse anti-HMGB1 antibody. BSA and β-tubulin were used as loading controls for the conditioned medium and the cytosolic proteins, respectively. (A) A group of representative Western blots; (B and C) Statistical analysis of HMGB1 protein levels in the conditioned medium (B) and cytosolic extracts (C) Data shown were mean ± SD from 4 DLBCL cell lines. ‘***’ in (B) indicates significant difference of HMGB1 levels in the conditioned medium between R-CHOP and CHOP treated samples. **D.** HMGB1 translocation. Cells were treated with rituximab alone for 4 hours. Fixed/Permeabilized cells were co-stained with HMGB1 antibody showing red and DAPI (blue) for nuclear localization. **E.** GA-101 antibody-induced HMGB1 release. Cells were incubated with or without 10 μg/ml GA-101 antibody for 4 hours. HMGB1 in the conditioned medium was determined by Western blotting. **F.** Anti-T cell antibody does not induce HMGB1 release in malignant B-cells. Su-6 and Su-8 cell lines were treated with 10 μg/ml anti-CD3 (OKT3) antibody for 4 hours. Rituximab (R)-treated cells were used as a positive control. (G and H) Comparison of the patterns between HMGB1 and cathepsin D release. **G.** DoHH2 cells were treated with rituximab for 4 hours. Fixed/Permeabilized cells were co-stained with anti-HMGB1 (red) and anti-cathepsin D (green) antibodies, and DAPI (blue). Arrow shows the colocalization of HMGB1 and cathepsin D. The single color images were shown in the Supplemental Figure 2A. **H.** Colocalization analysis. PDM images and correlation coefficient (Rr) were generated by the intensive correlation analysis using ImageJ software. The orange color and higher Rr indicate a colocalization and the blue color and a lower or negative Rr means a segregation.

Using Western blotting, we detected that R-CHOP but not CHOP induced a significantly increased HMGB1 release from DLBCL cells after treatment for 4 hours, without inducing changes in the levels of HMGB1 expression in these cell lines. CHOP neither induced nor enhanced rituximab-mediated HMGB1 release (Figure 2A–C and Supplemental Figure 1B). We monitored rituximab-induced HMGB1 intracellular shuttling using fluorescent microscopy. In untreated DLBCL cells, HMGB1 mainly localizes in the nucleus, although a weak expression of cytoplasmic HMGB1 can be detected. After treatment with rituximab alone for 4 hours, a strong staining of HMGB1 appeared in the cytoplasm (Figure 2D). Using Western blotting, we confirmed that both GA-101 (type-II anti-CD20 antibody) and rituximab induced HMGB1 release from DLBCL cells after treatment for 4 hours. An anti-CD3 antibody, OKT3 could not induce HMGB1 release on DLBCL cells (Figure 2E and 2F), indicating that induction of HMGB1 release is not a function of generic antibodies.

It was reported that HMGB1 release from monocytes is associated with lysosome exocytosis [30]. Coincidently, other study found that anti-CD20 antibodies induce lysosomal permeability in malignant B-cells [10, 14]. We therefore tested whether rituximab-induced the release of HMGB1 and cathepsin D, a lysosomal enzyme, follow the same pattern. Results from fluorescent microscopy and colocalization analysis showed that HMGB1 and cathepsin D shared colocalization in untreated DLBCL cells, as shown the yellow dots in Figure 2G and Supplmental Figure 2A and orange pixels (pseudo-colored) in Figure 2H. However, the Rr values decreased from 0.874 to 0.406 after treatment with rituximab (Figure 2H), indicating a decreased colocalization. We further co-stained cathepsin D or HMGB1 with cytoplasmic S6 ribosomal protein. Rituximab-induced cathepsin D release from the lysosome was restricted inside the cell (Supplemental Figure 2B) but HMGB1 was released to the outside (Supplemental Figure 2C). Using Western blotting, we confirmed that treatment with rituximab alone induced a release of HMGB1 but not cathepsin D (Supplemental Figure 2D). Taken together, our results demonstrate for the first time that rituximab induces HMGB1 release from live malignant B-cells and this release is unlikely via the mechanism of lysosomal exocytosis.

### Rituximab-induced HMGB1 release and STAT3 inhibition are coupled events

**Figure 3:**
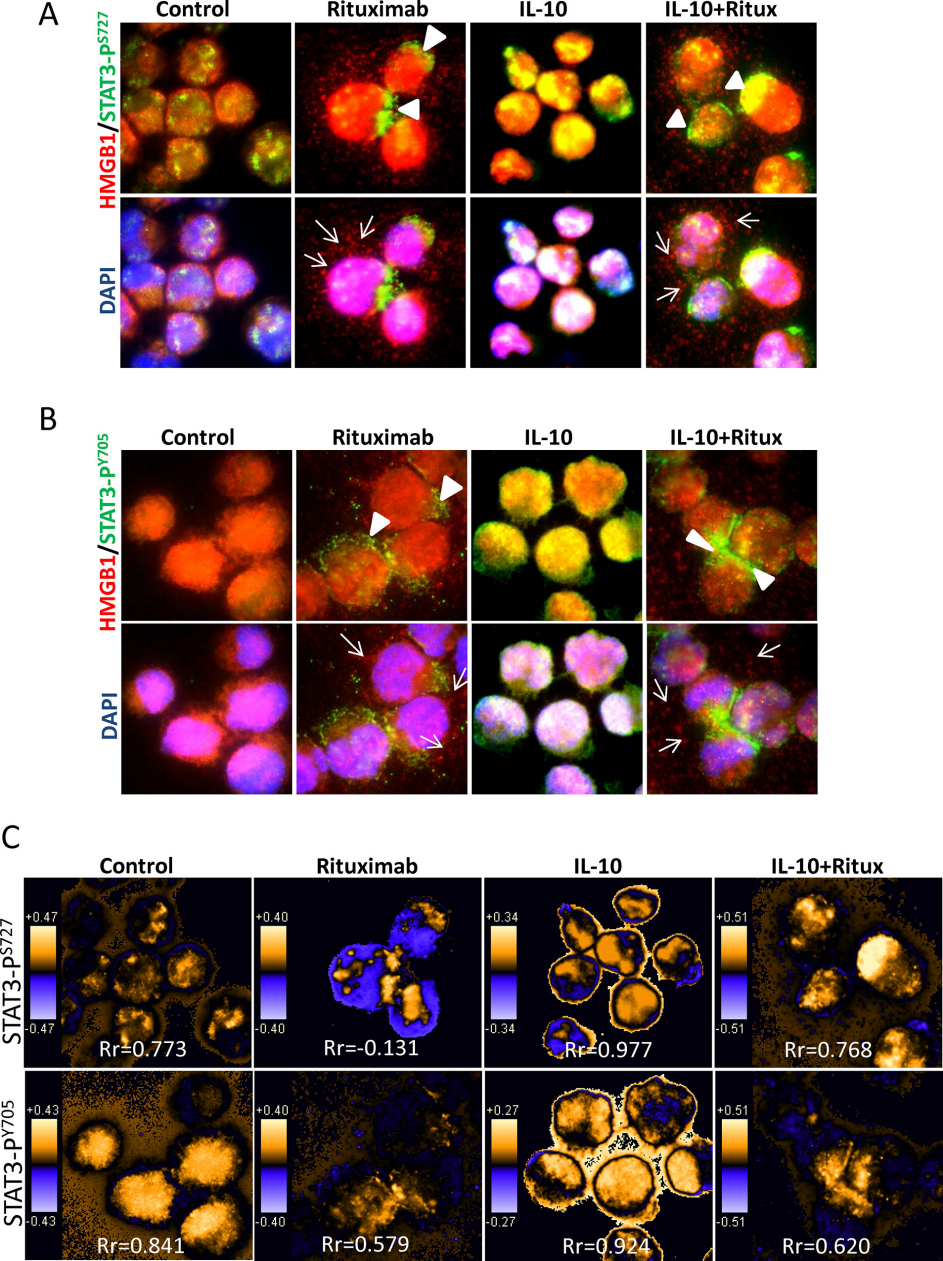
Rituximab induced intracellular shuttling of STAT3 and HMGB1 DoHH2 cells were treated with rituximab for 4 hours. Cells were co-stained with a mouse anti-HMGB1 (red) and a rabbit anti-STAT3-P^S727^ **A.** or STAT3-P^Y705^ **B.** antibodies (green). The arrow head shows the active STAT3 shuttled to the cytoplasm and the arrow indicates HMGB1 release from the cell. The single color images were shown in the Supplemental Figure 3. **C.** Colocalization analysis. PDM images and correlation coefficient (Rr) were generated by the intensive correlation analysis using WCIF ImageJ software. The orange color and higher Rr indicate a colocalization and the blue color and a lower or negative Rr means a segregation.

The function of HMGB1 in the nucleus is to help transcription factors and other nuclear proteins bind to their cognate sites by bending the DNA molecule [31]. One of the cytotoxic functions of rituximab is to inhibit the activity of transcription factor STAT3 [12]. However, the functional link between STAT3 and HMGB1 is unknown. We therefore tested whether rituximab-induced HMGB1 release is associated with the inhibition of STAT3. DLBCL cells were treated with rituximab and/or IL-10, a stimulator for STAT3, and then co-stained with HMGB1 (red) and either STAT3-P^S727^ or STAT3-P^Y705^ (green) antibodies (Figure 3A and 3B and Supplemental Figure 3). In control cells, most STAT3 and HMGB1 were localized in the nucleus, although weakly expressed in the cytoplasm. Treatment with IL-10 stabilized nuclear localization of both STAT3 and HMGB1. Rituximab induced a shuttling of both STAT3/HMGB1 to the cytoplasm and HMGB1 release, even in the presence of IL-10. Using colocalization analysis, it was shown that IL-10 enhanced but rituximab decreased STAT3 and HMGB1 nuclear colocalization (Figure 3C).

**Figure 4:**
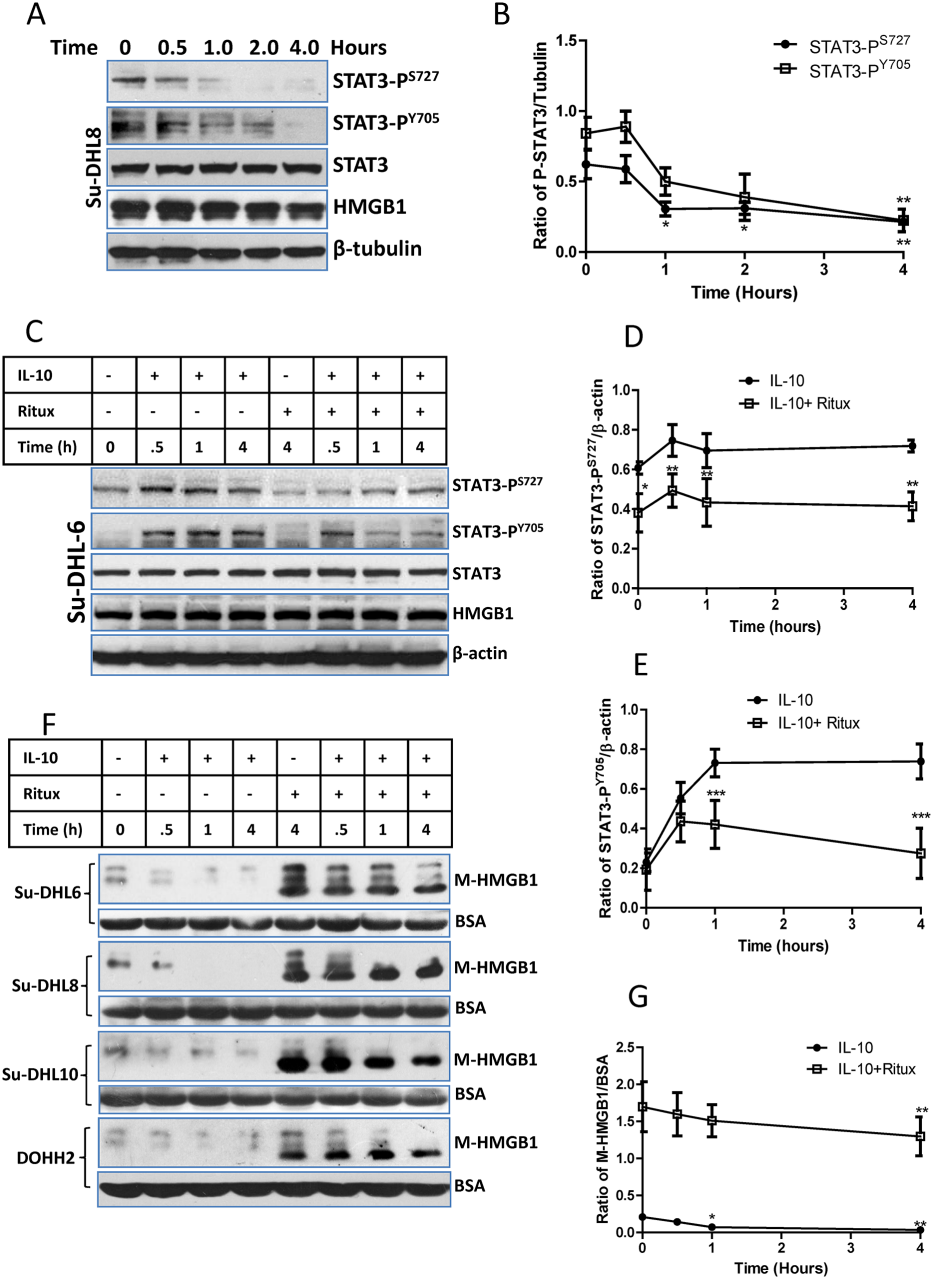
Correlation between STAT3 inhibition and HMGB1 release **A** and **B.** Rituximab-induced a time-dependent inhibition on STAT3 activity. (A) A group of representative Western blots and other groups of Western blots were presented in the Supplemental Figure 4A; (B) Statistical analysis of rituximab induced STAT3 inhibition. Data shown were mean ± SD of STAT3/β-tubulin from 4 different cell lines. Rituximab-induced significantly decreased STAT3 activities were compared with their controls using two-way ANOVA analysis. **C, D** and **E.** rituximab-induced STAT3 inhibition on IL-10 treated cells. DLBCL cells were treated with 10 ng/ml of IL10 and/or 10 μg/ml of rituximab simultaneously. (C) A group of representative Western blots and other groups of Western blots were presented in the Supplemental Figure 4B; (D and E) Statistical analysis of rituximab-induced inhibition on STAT3-P^S727^ and (D) STAT3-P^Y705^ (E) Data shown were mean ± SD from four different cell lines. Rituximab-induced significant inhibition of STAT3 activities on IL-10-treated cells were compared with those treated with IL-10 alone using two-way ANOVA analysis. **F** and **G.** Effects of IL-10 and rituximab on HMGB1 release. After cells were treated with IL-10 and/or rituximab, the conditioned medium was used for Western blotting. (F) Western blotting detection of HMGB1 in the conditioned medium (M-HMGB1). BSA was used as a loading control. (G) Statistical analysis of IL-10 and/or rituximab on HMGB1 release. Data shown were mean ± SD of M-HMGB1/BSA from four DLBCL cell lines. IL-10 significantly inhibited HMGB1 release was compared with the control.

The correlation between STAT3 activity and HMGB1 release were quantitatively analyzed by Western blotting in four DLBCL cell lines. First, we found that rituximab significantly inhibited STAT3 activity in a time-dependent manor (Figure 4A and 4B and Supplemental Figure 4A) and it also significantly inhibited IL-10-mediated STAT3 activation (Figure 4C–E and Supplemental Figure 4B). Unsurprisingly, IL-10 inhibited HMGB1 release from both control and rituximab-treated cells (Figure 4F and 4G). The association between STAT3 activity and HMGB1 release were analyzed using Pearson's correlation method in combination of data from Figure 4D, 4E and 4G. Statistical data (Supplemental Figure 5A and 5B) showed that the activities of both STAT3-P^S727^ and STAT3-P^Y705^ were significantly and negatively correlated with levels of extracellular HMGB1 (*P* < 0.01; γ = −0.88 and −0.89).

**Figure 5:**
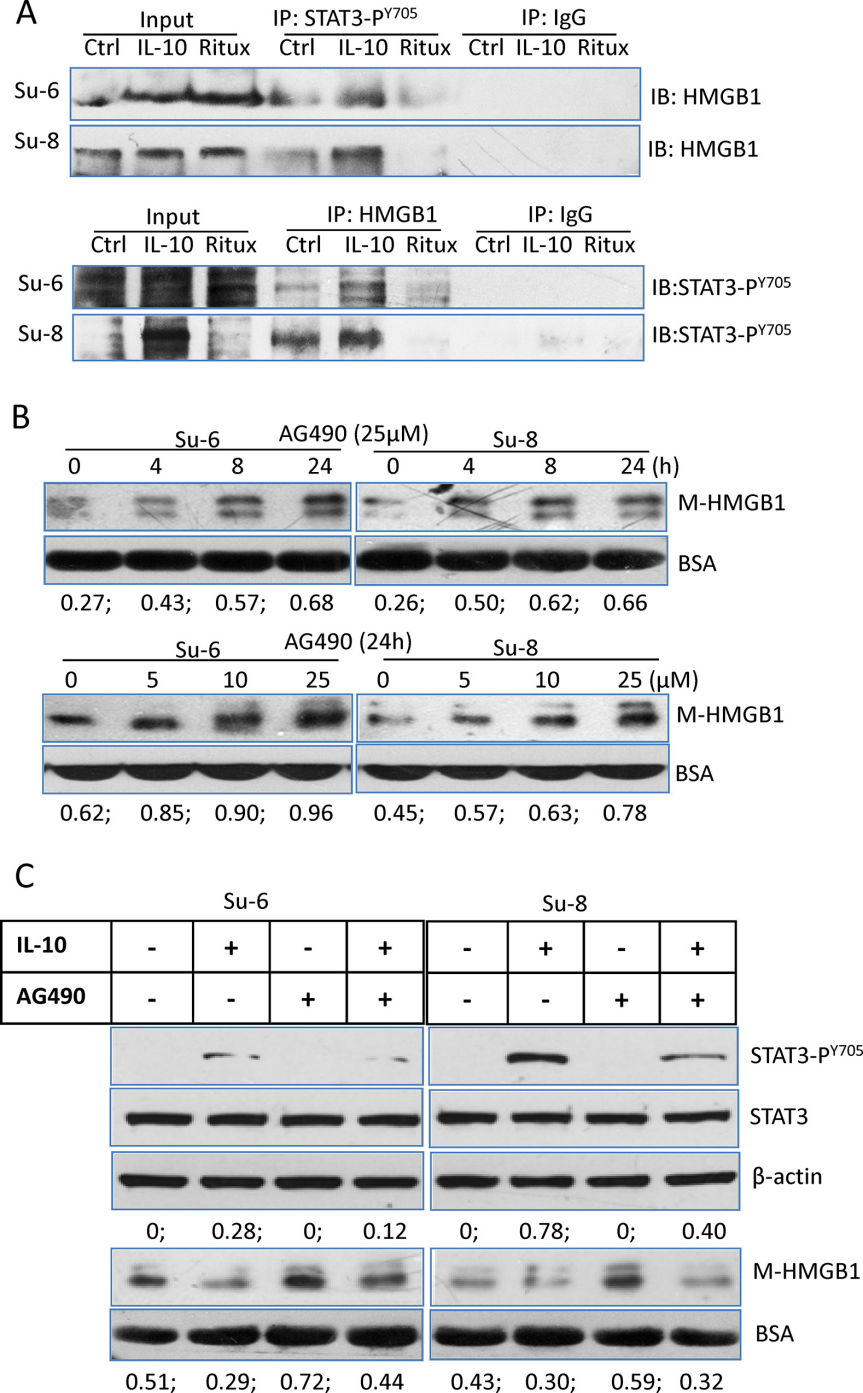
Association between STAT3 and HMGB1 **A.** Protein binding between HMGB1 and STAT3-P^Y705^. Su-6 and Su-8 cell lines were treated with either IL-10 or HMGB1 for 4 hours. Proteins from cell extracts were precipitated with either a rabbit anti-STAT3-P^Y705^ or a rabbit anti-HMGB1 antibodies. Rabbit IgG was used as a negative control. After Co-IP, blots were probed with a mouse anti-HMGB1 or a mouse anti-STAT3-P^Y705^ antibodies, respectively. **B.** AG490-induced time- and dose-dependent HMGB1 release. Cells were either treated with 25 μM AG490 up to 24 hours or with different doses of AG490 for 24 hours. HMGB1 in the conditioned medium was determined by Western blotting. Numbers under each blot are ratios of M-HMGB1/BSA. **C.** Roles of IL-10 and AG490 on STAT3 activity and HMGB1 release. DLBCL cells were treated with IL-10 and or AG490 for 4 hours. Expression of STAT3-P^Y705^ in the cell lysates and HMGB1 levels in the conditioned medium were determined by Western blotting.

To further confirm the association between STAT3 and HMGB1, we conducted co-IP to determine the binding between two proteins using either anti-HMGB1 or anti-STAT3-P^Y705^ antibody. There was a weak binding between STAT3 and HMGB1 in the control cells. The levels of binding were increased or decreased upon treatment with either IL-10 or rituximab (Figure 5A), demonstrating that rituximab reduced the binding between two proteins. Importantly, AG490, an inhibitor of JAK2/STAT3, induced HMGB1 release in a dose and time-dependent manor (Figure 5B). AG490-induced HMGB1 release can be inhibited by IL-10 (Figure 5C). Taken together, these results demonstrate that inhibition of STAT3 by rituximab breaks the binding between STAT3 and HMGB1. This could be the cause of HMGB1 release.

### Plasma from R-CHOP-treated patients show significantly increased HMGB1 and decreased IL-10

**Figure 6:**
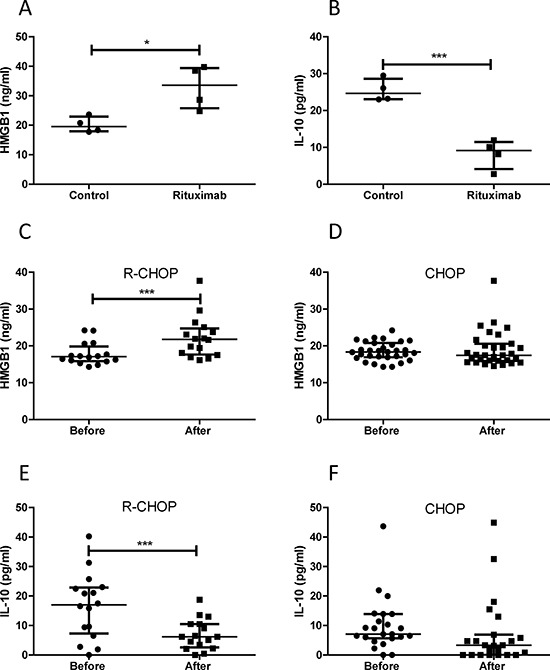
Quantitative analysis of HMGB1 and IL-10 levels **A** and **B.** Rituximab induced HMGB1 release and inhibition on IL-10 production in DLBCL cell lines. Four DLBCL cell lines in 2 × 10^6^/ml concentration were treated with rituximab for 4 hours. 10 μl of conditioned medium was used to measure concentrations of HMGB1 (A) and IL-10 (B) **C** and **D.** HMGB1 plasma concentrations before and after treatment with R-CHOP (C) or CHOP (D) **E** and **F.** IL-10 plasma concentrations before and after treatment with R-CHOP (E) or CHOP (F) Data shown are median ± interquartile range. Two pairs of extreme data, HMGB1 (from 135.38 to 25.67 ng/ml) and IL-10 (from 432.45 to 458.97 pg/ml) before and after treatment with CHOP were removed from (D) and (F), respectively, for presentation purpose.

Using ELISA kits, we quantitatively confirmed that rituximab induced a significantly increased HMGB1 release and a significantly decreased IL-10 secretion in DLBCL cell lines (Figure 6A and 6B). To determine whether treatment with rituximab induces HMGB1 release and STAT3 inhibition *in vivo*, we compared HMGB1 and IL-10 concentrations in the plasma of DLBCL patients before and after treatment with CHOP (*n* = 24) and R-CHOP (*n* = 16). The median levels of HMGB1 and IL-10 in DLBCL patients plasma were 17.88 ng/ml and 9.49 pg/ml, respectively, and were higher than previously published normal levels of HMGB1 <10 ng/ml [32] and IL-10 <3 pg/ml [33]. After treatment with R-CHOP, HMGB1 levels in the plasma were significantly increased (Figure 6C) and IL-10 levels were significantly decreased (Figure 6E) compared with their untreated samples. However, treatment with CHOP did not affect the plasma levels of HMGB1 and IL-10 (Figure 6D and 6F). These results further confirmed that treatment with rituximab induces STAT3 inhibition and HMGB1 release from DLBCL B-cells.

### Treatment with rituximab elicits an immune response

Extracellular HMGB1 is one of the ICD markers. We tested whether treatment with rituximab elicits other ICD markers, such as CRT exposure and ATP release. Treatment with CHOP or rituximab alone increased phosphorylation of eIF2α (P-eIF2α), one of the precursors of CRT exposure (Supplemental Figure 6A). However, rituximab induced an overexpression of CRT protein but did not induce CRT surface exposure on DLBCL cells (Supplemental Figure 6A and 6B). In addition, neither rituximab nor GA101 induced ATP secretion (Supplemental Figure 7).

**Figure 7:**
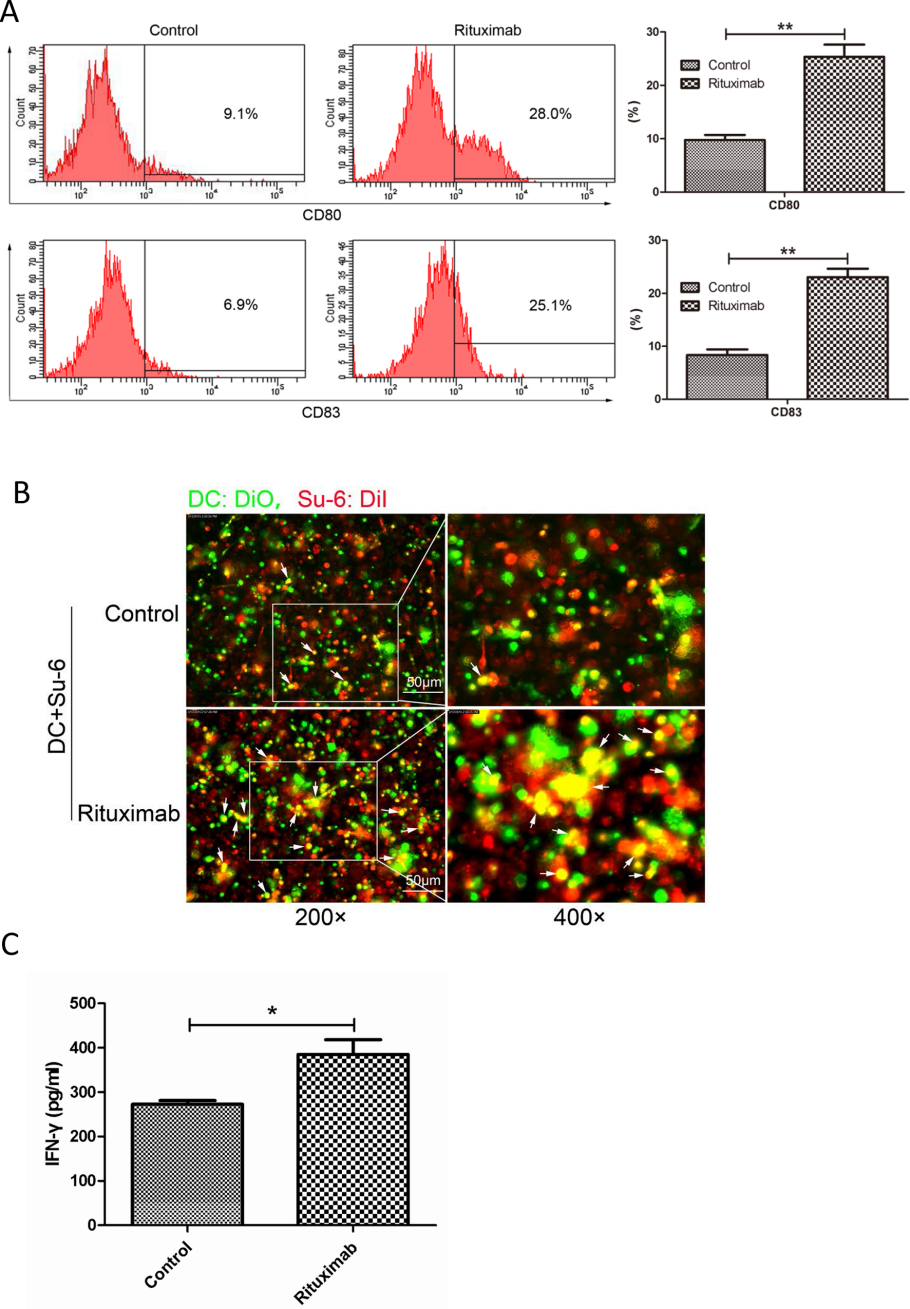
Rituximab induced immune response in both DCs and cytotoxic T-cells **A.** DC maturation. DCs were stimulated with conditioned supernatant of control or rituximab-treated cell culture medium. The maturation markers for DCs, CD83 or CD80, were evaluated by flow cytometry and compared between control and rituximab-treated groups. **P < 0.01. Data were expressed as mean ± SD and each value represented the mean of three replicates. **B.** Fluorescence microscopy analysis of phagocytosis. After 24 hours co-culture of immature DCs with rituximab-treated tumor cells, the engulfment of tumor cells was verified by fluorescence microscopy. DCs were stained with DiO (green) and Su-6 cells were stained with DiI (red). Double positive or yellow cells are fully phagocytized cells. **C.** IFN-γ production. Immature DCs were pulsed with rituximab-treated DLBCL cells for 24 hours and then co-cultured with autologous CD8^+^ T lymphocytes. IFN-γ concentration in the supernatant was assessed after 10 days using the IFN-γ ELISA kit.

We therefore tested whether HMGB1 in the conditioned medium from rituximab-treated cells could induce an immune response. Human immature dendritic cells (iDCs) (treated with GM-CSF and IL-4 for 5 days) were incubated with conditioned supernatants from rituximab treated cells for another 24 hours. Maturation of DCs was determined by expression of CD80 and CD83 using flow cytometry. Rituximab-treated cell culture medium induced significantly increased expression of both CD80 and CD83, indicating maturation of DCs (Figure 7A). To determine phagocytosis, iDCs (treated with GM-CSF and IL-4 for 5 days) were co-cultured with DLBCL cells treated with saline, or rituximab for 24 hours and then, fluorescence microscopy was used to evaluate the phagocytosis of DCs. DCs activated with rituximab-treated cells showed phagocytosis activity compared with the control, as shown by double positive, or yellow colored cells (Figure 7B). Moreover, the co-culture matured DCs with autologous CD8^+^ T cells significantly increased the secretion of IFN-γ by cytotoxic T-cells compared with the control (Figure 7C). These results demonstrate that conditioned supernatant from rituximab-treated DLBCL cells can enhance immune response by inducing maturation and phagocytosis of DCs and up-regulate cytotoxic function of T cells.

## MATERIALS AND METHODS

### Human DLBCL plasma

Plasma was collected from 40 DLBCL patients, (Supplemental Table 1) at diagnosis and after treatment with CHOP or R-CHOP for 2 weeks and stored at −80°C. DLBCL patients were diagnosed by standard criteria and treated in Tianjin Cancer Hospital, China during 2014. Ethical approval for this study was obtained from the ethics committee of Tianjin Medical University Cancer Institute and Hospital (Ethic number Ebc2014).

### Cell lines and cell culture

Human DLBCL cell lines Su-DHL6 (Su-6), Su-DHL8 (Su-8), and Su-DHL10 (Su-10) were kindly provided by Professor Anthony Letai (Dana-Farber Cancer Institute) [34, 35] and the DoHH2 cell line was obtained from CRUK tissue bank [36]. Cell lines were routinely cultured in RPMI-1640 medium supplemented with 10% heat-inactivated fetal calf serum (FCS) and 2.0 mM L-glutamine at 37°C in a 5%CO_2_ humidified incubator.

### Treatment with CHOP or R-CHOP

The components of CHOP, cyclophosphamide, doxorubicin, vincristine and prednisolone were purchased from Sigma. Rituximab (MabThera) and GA101 were obtained from Roche. The stock solutions for CHOP were prepared individually and then mixed together according to the ratios of the *in vivo* treatment [6], as listed in Supplemental Table 2. Cells were treated with CHOP, or R-CHOP (CHOP plus 10 μg/ml of rituximab) for 24 or 48 hours to induce cell death.

### Cell death and cytotoxicity assays

To determine cell death, cells were stained with 0.5 mg/ml of 7-AAD (KeyGen Biotech) for 10 min and 7-AAD positive (dead) cells were determined by flow cytometry (BD FACS Canto II, BD, USA). CHOP or R-CHOP-induced cytotoxicity was assessed by Cell Counting Kit-8 (CCK-8, Beyotime Biotechnology) according to the manufacturer's instruction. Briefly, 5000 cells in 100 μl were seeded in each well in a 96-well plate. After treatment with CHOP or R-CHOP for 48 hours, 10 μl of CCK-8 solution was added into each well and cells were further incubated for 1 hour. The OD values were determined by a plate reader at the wavelength of 450nm.

### Western blotting analysis

Whole-cell extracts were prepared by lysing cells with the RIPA lysis buffer supplemented with protease inhibitor and phosphatase inhibitor cocktails (Sigma). Cellular protein lysates (20 μg) were separated by 10% SDS-PAGE, and target proteins were detected by Western blotting, as described previously [37]. Antibodies were listed in Supplemental Table 3 and 4.

### Determination of ICD markers

HMGB1 release was qualitatively and quantitatively determined by Western blotting and ELISA, respectively [32]. To determine CRT exposure, the cells were fixed with 0.25% of paraformaldehyde in PBS for 10 min and blocked with 3% bovine serum albumin (BSA). After stained with PE-conjugated anti-CRT antibody for 30 min, PE-positive cells were determined by flow cytometry. ATP concentration in the culture medium was performed using an ATP Determination Kit (Life technologies) according to the manufacturer's instructions [37].

### Preparation of human DCs and CD8+ T lymphocytes

Human peripheral blood mononuclear cells (PBMCs) were isolated from healthy donors by Ficoll-Hypaque density gradient centrifugation and cultured in RPMI 1640 medium containing 10% FCS for 2 hours. DCs, which were generated from the adherent fraction of PBMCs [38], were cultured for 5 days in RPMI 1640 medium containing 10% FCS, 20 ng/mL human GM-CSF, and 10 ng/mL human IL-4 (Biolegend). Culture medium and cytokines were refreshed in every other day. On the day 5, the medium was replaced by rituximab-treated conditioned medium or control medium and cell culture was continued for 24 hours. Autologous CD8+ T lymphocytes were magnetically isolated using CD8 MicroBeads (Miltenyi Biotech, Germany) to obtain purity ≥95% CD8+ T cells. The isolated T lymphocytes were cultured in RPMI 1640 medium containing 10% FCS and 10 ng/mL human IL-2 (Biolegend) and the medium was replaced every other day [37].

### Immuno-staining and fluorescent microscopy

Cells on slides were fixed and permeabilized with Cytofix/Cytoperm reagents (BD) and blocked with a buffer consisting of 0.1% saponin and 5% serum (the type of serum corresponding to the isotype of the secondary antibody). Cells were co-stained with a monoclonal and a polyclonal primary antibody (Supplemental Table 3) for 1 hour at room temperature. After washing with TBST (TBS containing 0.1% Tween-20), cells were incubated with Alexa-Fluor conjugated secondary antibodies (Supplemental Table 4) at 1:100 dilution. Slides were washed for 3 times with TBST, stained with 50 ng/ml DAPI (4′, 6-diamidino-2-phenylindole, Sigma), air-dried at 4°C in the dark, and mounted in ProLong^®^ Gold anti-fade reagent (Life Technologies) before being viewed under Nickon Intensilight C-HGFI fluorescent microscope (Nickon, Japan) [32].

The anti-tumor phagocytic function of matured DCs was assessed after DCs or DLBCL cells were labelled fluorescently with *Vybrant*^™^ DiO (V-22886) or DiI (V-22885) cell-labelling solutions (Life technologies), respectively. Briefly, DLBCL cells were treated with rituximab for 4 hours after stained with DiI (1:200 dilution). DiO-loaded immature DCs (Day 5) were then incubated with the rituximab-treated cells. After co-culture for 24 hours, the cells were fixed with 4% paraformaldehyde for 20 min, washed in PBS for 20 min and mounted on slides and viewed under a fluorescent microscope. Double positive cells were considered as fully phagocytozed cells [37].

### Colocalization analysis

Colocalization analysis was based on the theory that if two proteins are parts of the same complex, then their staining intensities should vary in synchrony, whereas if they are on different complexes or structures, they will exhibit asynchronous staining. The intensive correlation analysis (ICA) method was used for determine the levels of colocalization. Image of (PDM): the Product of the Differences from the Mean was used to qualitative analysis of colocalization. The levels of colocalization were quantitatively expressed by the Pearson's correlation coefficient (Rr) using WCIF ImageJ software [39–41].

### Co-immuno-precipitation (Co-IP)

Cell extracts were prepared with the RIPA buffer. 200 μg Proteins were incubated at 4°C overnight with a rabbit anti-STAT3-P^Y705^, a mouse anti-HMGB1 antibody, or a control IgG. The immune complexes were immuno-precipitated on Protein A/G Plus-Agarose beads (Santa Cruz), washed for 3 times with TBST, resolved on 10% SDS gel, and detected with anti-HMGB1 or anti-STAT3-P^Y705^ antibody, respectively.

### ELISA quantitative analysis

Concentrations of HMGB1 or IL-10 in human plasma or conditioned medium were determined by HMGB1 ELISA kit (IBL International) [32] or IL-10 ELISA kit (Cloud-Clone Corp.), respectively. IFN-γ secretion from CD8+ T lymphocytes was assessed by IFN-γ ELISA Kit (Cloud-Clone Corp) according to the manufacturer's instructions [37].

### Statistical analysis

Statistical analysis was performed using GraphPad Prism software (version 5.03). For all experiments, at least 3 independent experiments were performed. Data are expressed as mean ± SD or median with interquartile range when variation was high. Significant difference between two groups with equal numbers was analyzed by two-sided Student *t*-tests and those with unequal sizes were analyzed with the Mann-Whitney *U* test. Two-way ANOVA with Bonferroni post-hoc test was used to compare difference between two groups of data. Correlation between two groups of variables was analyzed with Pearson's correlation. All *P*-values < 0.05 were considered as statistically significant. *, **, and *** indicate *P* value < 0.05, 0.001, and 0.0001, respectively.

## DISCUSSION

There is accumulating evidence for the hypothesis that long-term clinical success of anticancer therapy requires the participation of immune responses to ‘danger’ signals emitted by cancer cells [42, 43]. Dysregulated immune response confers a worse clinical outcome in DLBCL patients treated with R-CHOP [44, 45]. Our present data demonstrate for the first time that rituximab induces a cell death-independent HMGB1 release which in turn elicits cytotoxic immune responses. We therefore propose that rituximab may eliminate malignant B-cells via inducing immune responses rather than a direct killing.

Treatment with R-CHOP greatly improves overall survival rates in patients with DLBCL compared with CHOP. However, our *in vitro* experiments showed that treatment with R-CHOP does not kill more DLBCL B-cells compared with treating with CHOP alone. Rituximab did not directly induce cell death as tested in four different DLBCL cell lines. We hypothesized that rituximab may kill malignant B-cells indirectly and this led us to determine whether rituximab elicits ICD markers on malignant B-cells.

We found that rituximab induced HMGB1 release from intact DLBCL cells. Conversely, treatment with CHOP induced cell death but not HMGB1 release. According to the concept of ICD, HMGB1 is released from dying or dead cells and it is a late event of ICD [18]. Our previous study showed that plasma from chronic lymphocytic leukemia (CLL) patients has elevated levels of HMGB1 and the concentration of HMGB1 is positively correlated with the absolute lymphocyte count, suggesting that the plasma HMGB1 is released from live CLL B-cells [32]. However, this is not a phenomenon of malignant B-cells, because other live tumor cells, such as colon, gastric and liver cancer cells are also able to secret HMGB1 [46]. Treatment with R-CHOP increased plasma HMGB1 concentration in patients with DLBCL. Two of CHOP's components, doxorubicin and cyclophosphamide, have been found as ICD inducers [47]. However, treatment with CHOP did not induce HMGB1 release, CRT exposure and ATP secretion. This may be caused by the drug combination which lowers the dose of each drug. *In vitro* experiments showed that rituximab-induced HMGB1 release was detected as early as 1 hour and co-treatment with CHOP did not enhance HMGB1 release. We propose that rituximab-mediated HMGB1 release is unique and cell death-independent process compared with ICD inducers.

The mechanism of HMGB1 release from a cell is poorly understood. An early study demonstrated that HMGB1 secretion from monocytes is via lysosome exocytosis [30]. Our results showed that rituximab induces lysosome permeability but not lysosome exocytosis because HMGB1 release was not accompanied by the release of lysosomal enzyme cathepsin D. We believe that it will be a challenge to find out how HMGB1 is released from live cells.

STAT3 is constitutively activated in DLBCL cells [48]. It was reported that rituximab is able to inhibit STAT3 activity and IL-10 secretion by a CD20 positive Burkitt's lymphoma cell line, 2F7 [12]. Indeed, we confirmed that treatment with rituximab significantly inhibited the expression of both STAT3-P^S727^ and STAT3-P^Y705^, and secretion of IL-10 in a panel of DLBCL cell lines. We also found that plasma IL-10 levels were significantly decreased in R-CHOP treated DLBCL patients. IL-10 is an immunosuppressive cytokine [49]. Inhibition of IL-10 secretion may contribute, at least partly, to rituximab-induced immune response.

As a nuclear protein, HMGB1 binds to DNA and some of transcriptional factors, and it is required for gene transcription [50, 51]. We hypothesized that the active STAT3 may bind to HMGB1 and stabilize its nuclear localization. Inhibition of STAT3 by rituximab may break this binding and cause HMGB1 release from the nucleus. Our results confirmed that rituximab-induced STAT3 inhibition is significantly correlated with HMGB1 release. In contrast, stimulation of STAT3 by IL-10 stabilized HMGB1 nuclear localization. HMGB1 shares same localization with active STAT3 in the nucleus but segregation occurs when they translocate from the nucleus to the cytoplasm. Using co-IP, we detected the interaction between HMGB1 and the active STAT3. IL-10 and rituximab played either positive or negative roles on this interaction. To consolidate this finding, a JAK2/STAT3 inhibitor, AG490 was used to replace rituximab. As expected, AG490 induced both dose- and time-dependent HMGB1 release and this release is associated with an inhibition of STAT3 activity.

As rituximab neither induces cell death nor elicits other ICD markers, such as CRT exposure and ATP release, we propose that rituximab is unlikely an ICD inducer. Extracellular HMGB1 is known as one of the maturation factors for DCs [52]. Our results demonstrate that the conditioned medium from rituximab-treated DLBCL cells is able to induce maturation of DCs which in turn present tumor antigen to cytotoxic T cells. Rituximab alone could not induce maturation of DCs, suggesting that the immunogenic effect is not due to the activation of Fc receptor by rituximab.

It is currently unknown how rituximab-induced ICD is linked with previously proposed mechanism of ADCC. ICD is the killing mechanism by which DCs and T-cells are activated by tumor cell antigens and eventually tumor cells are killed by cytotoxic T-cells and phagocytic cells. ADCC is the natural killer cells kill antibody-primed tumor cells [53]. Both ICD and ADCC are immune-mediated tumor cell killing, by different mechanisms and different immune cells. Rituximab-mediated ICD can kill DLBCL cells and may also enhance rituximab-induced ADCC.

In summary, this study demonstrates that rituximab induces a cell death-independent HMGB1 release which is associated with STAT3 inhibition. Increased extracellular HMGB1 and suppressed IL-10 secretion elicit cytotoxic immune response which may eliminate malignant B-cells indirectly. Our data provide a new understanding on the mechanism by which rituximab and other anti-CD20 antibodies eliminate malignant B-cells.

## SUPPLEMENTARY FIGURES AND TABLES


