# Supplementary material for: Rituximab-induced HMGB1 release is associated with inhibition of STAT3 activity in human diffuse large B-cell lymphoma
Source: Oncotarget. 2015 Jul 31;6(29):27816–31. doi: 10.18632/oncotarget.4816 (PMC4695028; doi:10.18632/oncotarget.4816)
Supplement: Supplementary file 1 [file oncotarget-06-27816-s001.pdf]

SUPPLEMENTARY FIGURES AND TABLES

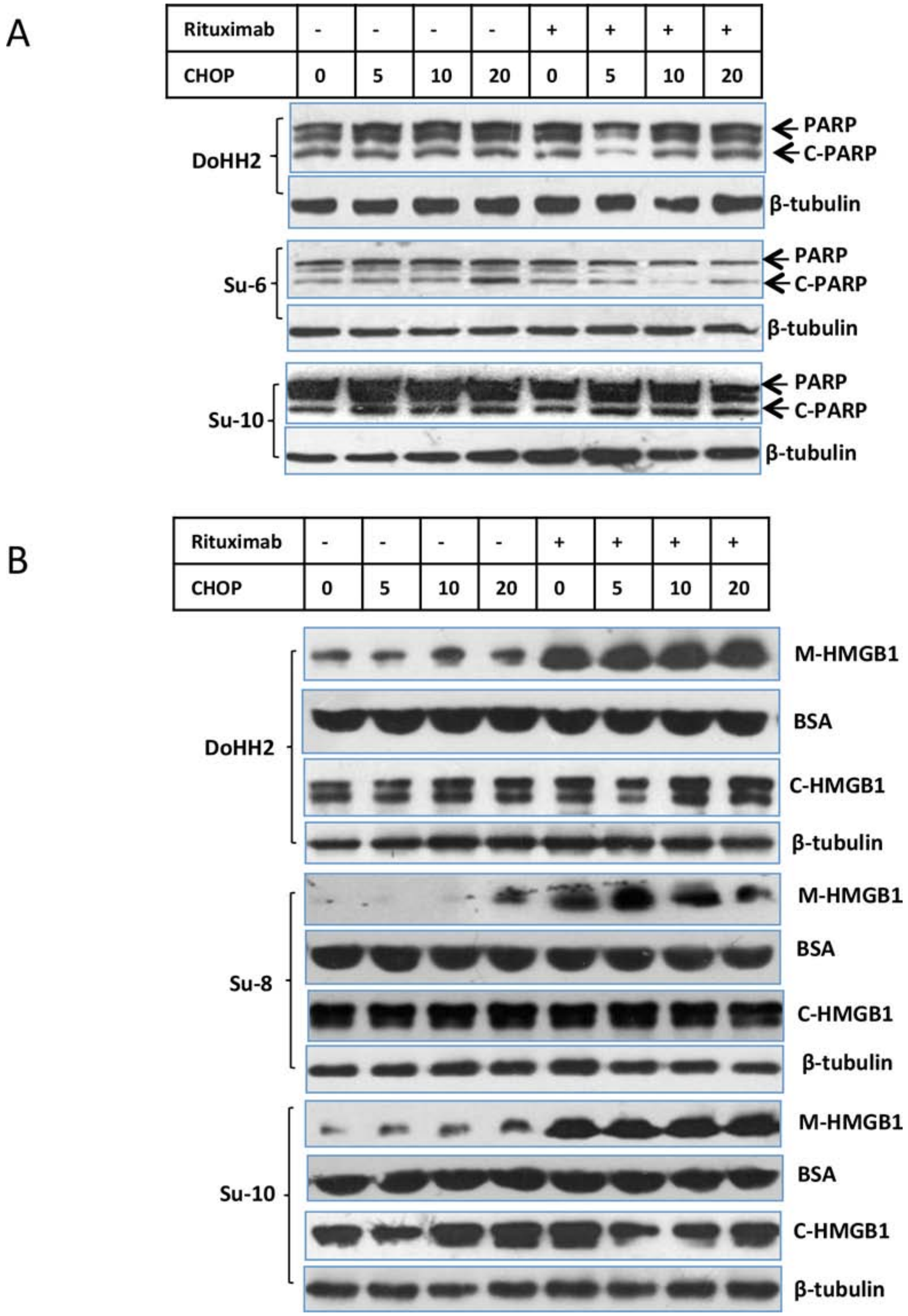

Supplementary Figure S1: Comparison of CHOP and R-CHOP-induced PARP cleavage and HMGB1 release. DLBCL cell lines were treated with CHOP or R-CHOP for 24 hours. Cytosolic proteins were used to test PARP Cleavage **A.** and HMGB1 expression (C-HMGB1) **B.** Conditioned medium (50 µl) was used to determine HMGB1 release (M-HMGB1) (B) BSA and β-tubulin were used as loading controls for conditioned medium Cytosolic proteins, respectively.

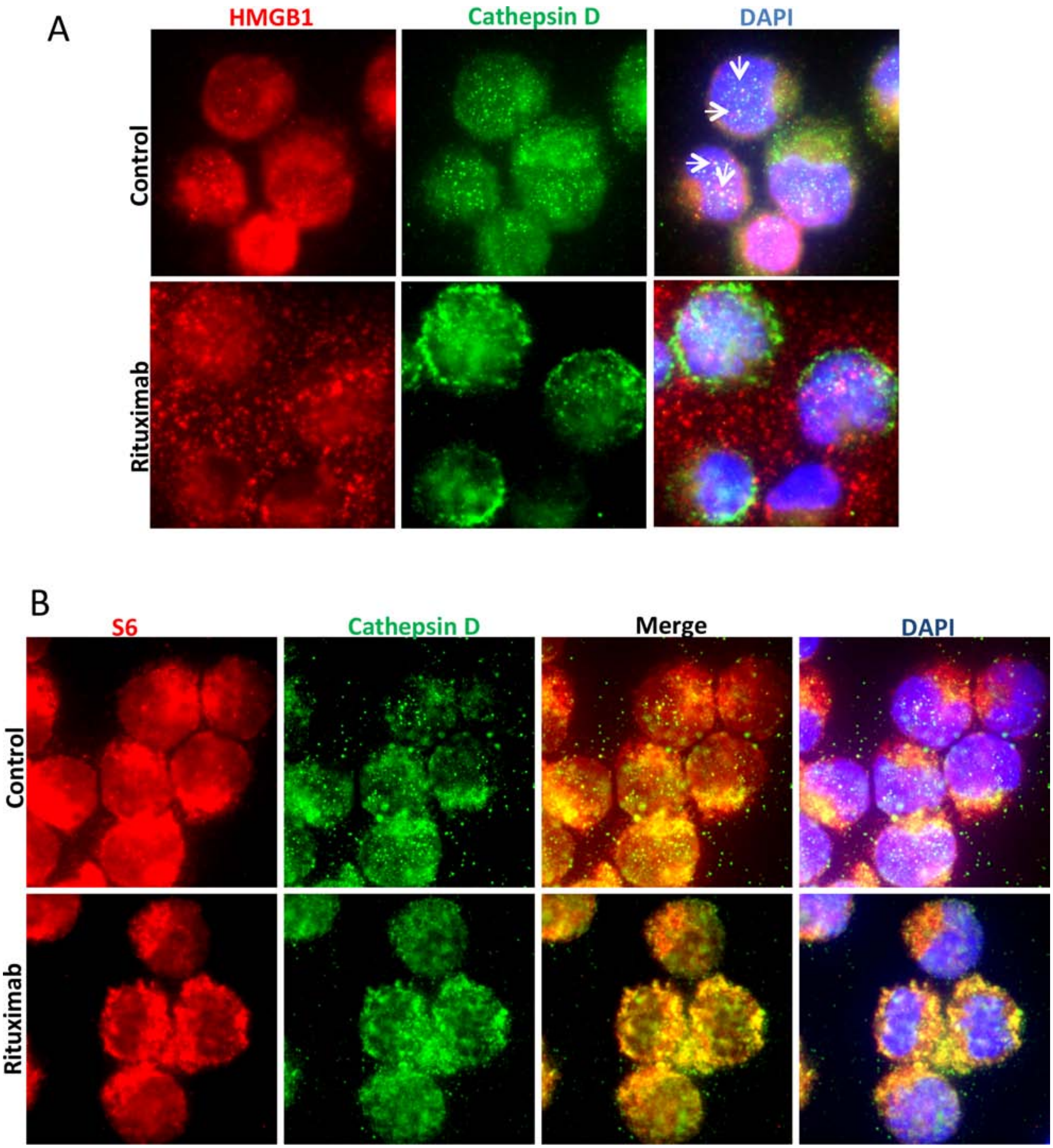

Supplementary Figure S2: The association between HMGB1 and cathepsin D release. DoHH2 cells were treated with Rituximab for 4 hours. Cells on slides were fixed/permeabilized and stained with rabbit anti-HMGB1/mouse anti-cathepsin D **A.** rabbit anti-S6/mouse anti-cathepsin D **B.** and rabbit anti-S6/mouse HMGB1

(*Continued*)

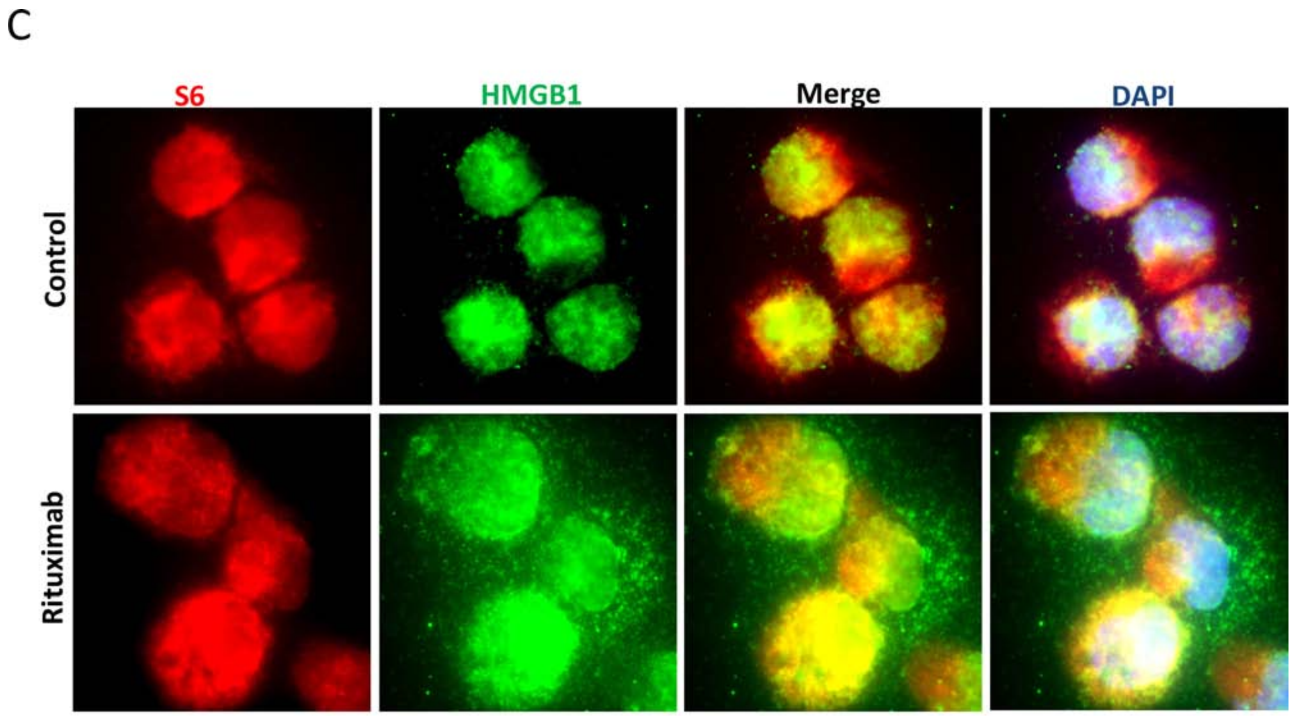

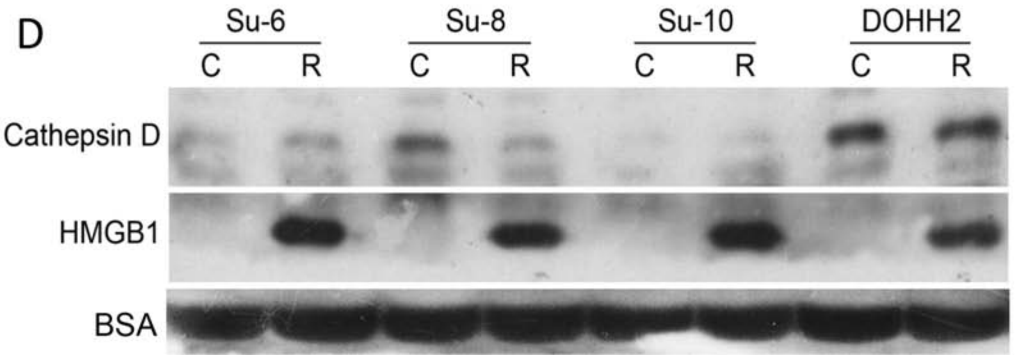

Supplementary Figure S2: (*Continued*) C, D. Four DLBCL cell lines were treated with rituximab for 4 hours. 50 μl of conditioned medium was used for Western Blotting. BSA was used as a loading control. 'C' means control and 'R' indicates Rituximab.

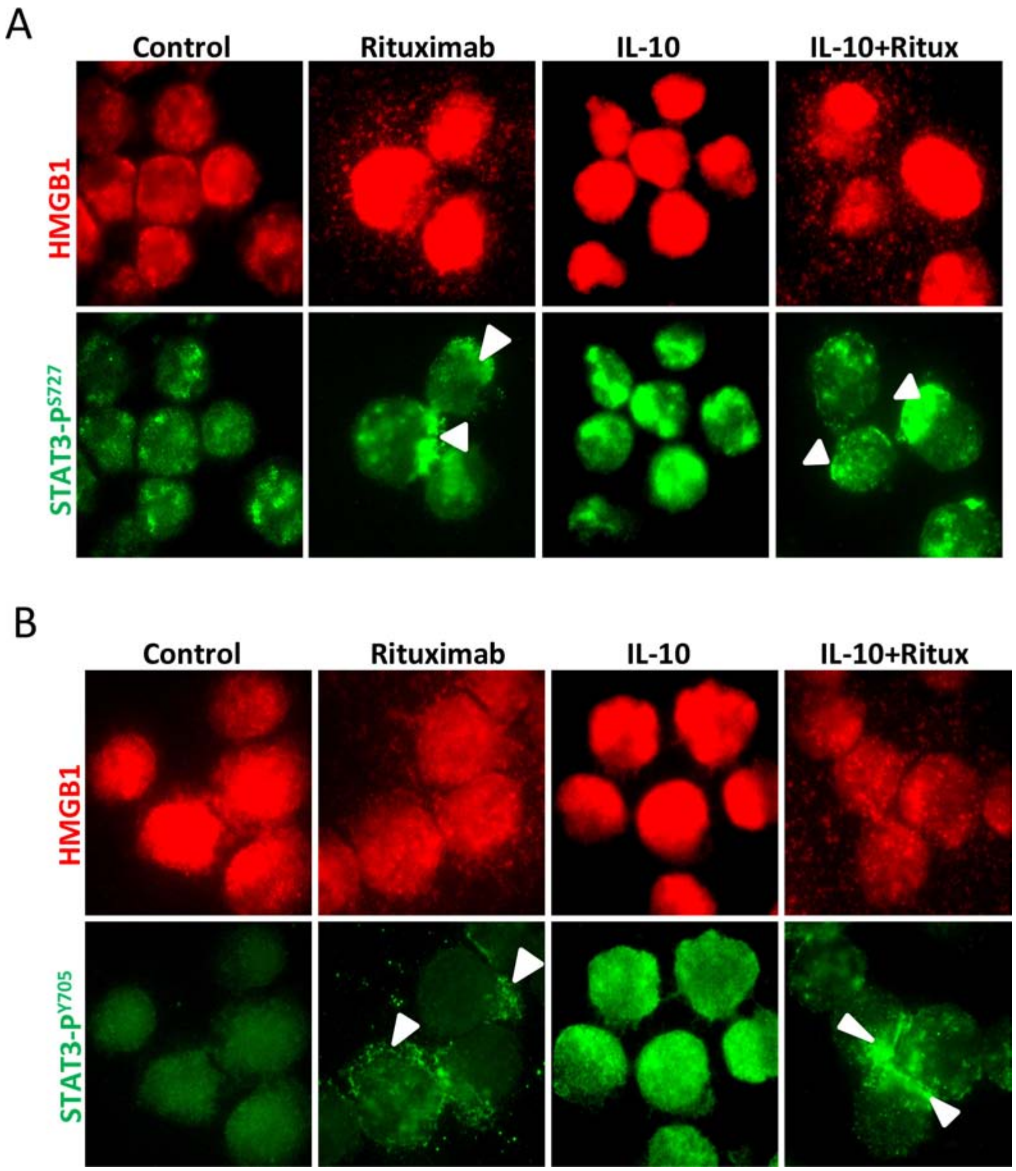

Supplementary Figure S3: Single color images of rituximab induced intracellular shuttling of STAT3 (green) and HMGB1 (red). A. STAT3-P^{S727} and **B.** STAT3-P^{Y705}. Arrow head indicate cytoplasmic localization of STAT3.

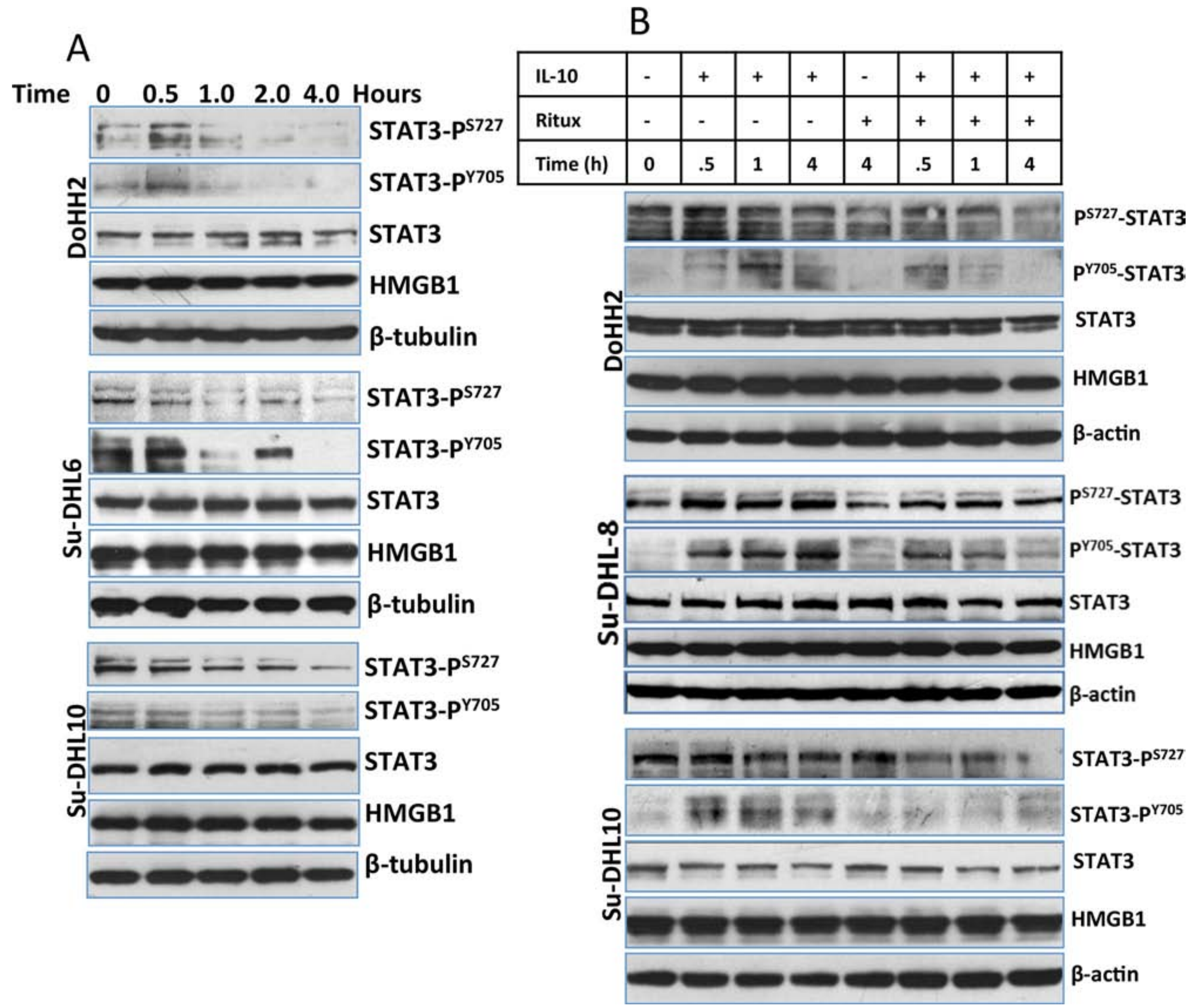

Supplementary Figure S4: Rituximab-induced STAT3 inhibition. A. Time dependent course of rituximab-induced inhibition of STAT3. **B.** Rituximab-induced STAT3 inhibition on IL-10 treated cells.

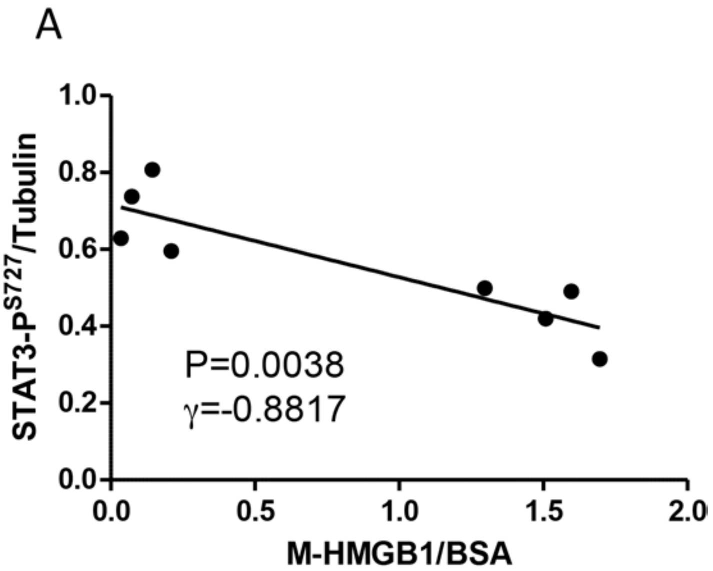

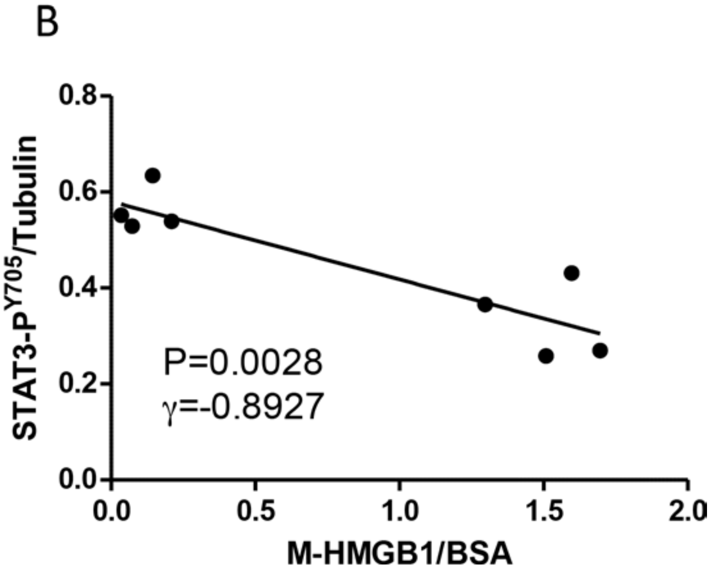

Supplementary Figure S5: Correlation between STAT3 activity and HMGB1 release. A. STAT3-P^{727}; **B.** STAT3-P^{Y705}. Data shown are mean values from Figure 4 D, 4E and 4G and analyzed by Pearson's Correlation using Prism Software.

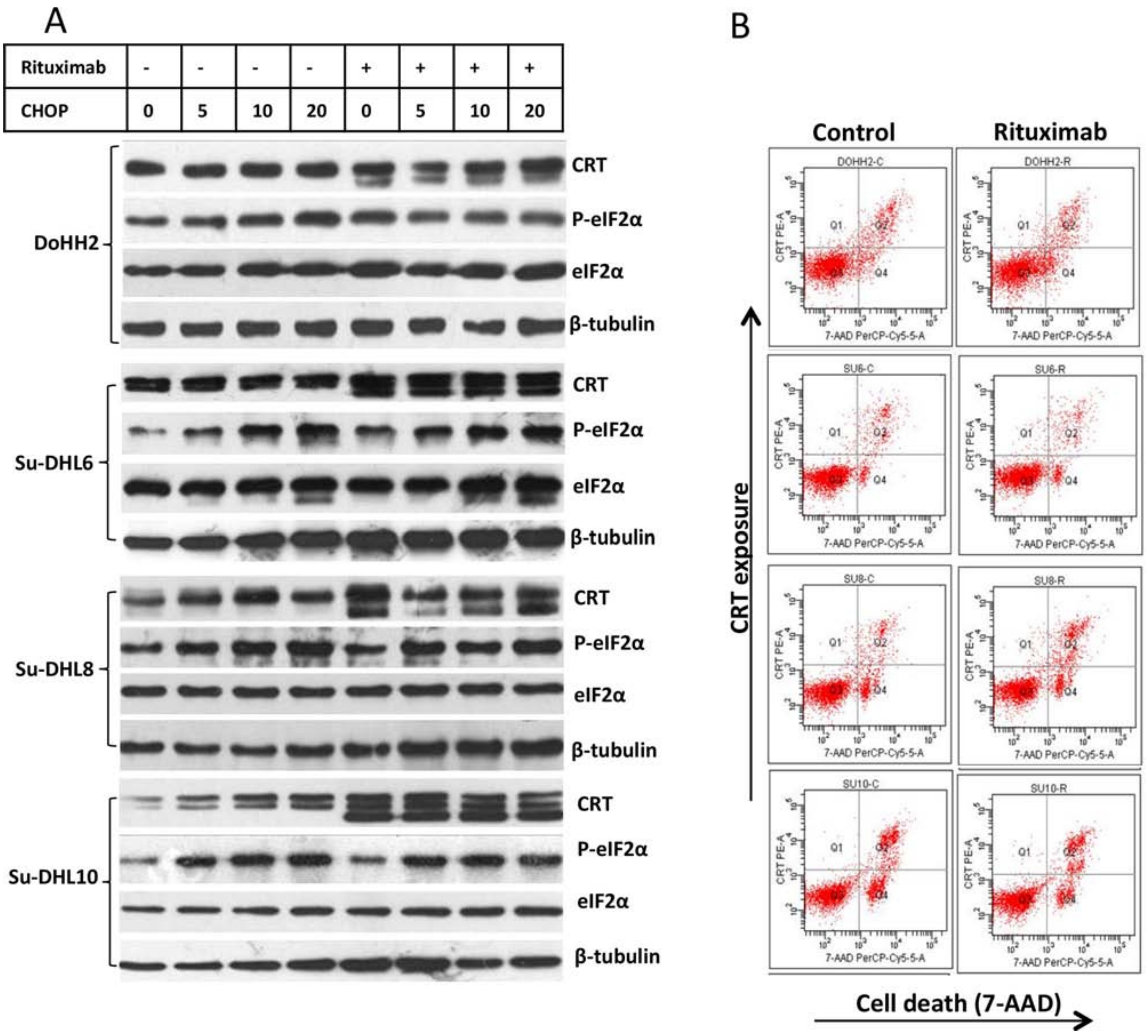

Supplementary Figure S6: Effect of CHOP or R-CHOP in inducing eIF2α phosphorylation (A) and CRT exposure (B). A. DLBCL cells were treated with CHOP or R-CHOP for 24 hours. Expression of CRT, P-eIF2α and P-eIF2α were determined by Western blotting. **B.** DLBCL cells were treated with rituximab for 4 hours. After fixation, cells were stained with CRT-PE and 7-AAD. Cells with CRT exposure were defined as 7-AAD negative and CRT-PE positive. We were not be able to identify CHOP-induced CRT exposure due to the auto-fluorescence of CHOP.

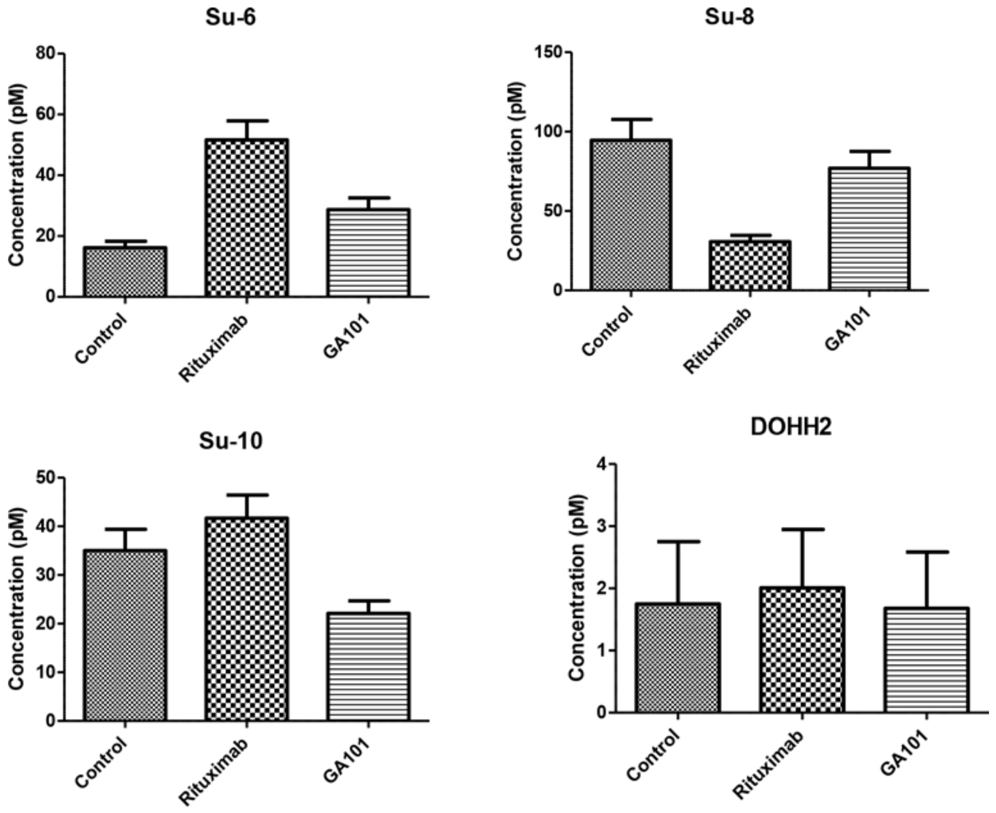

Supplementary Figure S7: Rituximab or GA101-induced ATP release. DLBCL cells were treated with 10 µg/ml of rituximab or GA101 for 4 hours. ATP concentration in the conditioned medium (10 *µl*) was determined using ATP chemiluminescence ELISA kit.

Supplementary Table S1: Summary of clinical characteristics of DLBCL patients' plasma used for ELISA

	No of patients	Percentage
**Total No**	40	100%
**Age**		
**>50**	26	65%
**<50**	14	35%
**Gender**		
**Male**	25	62.5%
**Female**	15	37.5%
**Stage at diagnosis**		
**I**	4	10%
**II**	18	45%
**III**	6	15%
**IV**	12	30%
**IPI score**		
**0**	11	27.5%
**1**	18	45%
**2**	7	17.5%
**3**	4	10%
**Treatment with**		
**CHOP**	24	60%
**R-CHOP**	16	40%
**Treatment response**		
**No response**	0	0%
**CHOP-PR**	24	100%*
**CHOP-CR**	0	0%*
**R-CHOP-PR**	12	75%**
**R-CHOP-CR**	4	25%**

Note: * indicates percentages of patients who were treated with CHOP and ** means percentages of patients who were treated with R-CHOP.

Supplementary Table S2: Preparation of CHOP

Drug (supplier)	Clinical Dose (mg/m^2)	Calculated ratio	Concentration* (mg/ml)	Amount of drug added to make CHOP, ml (mg)
**Cyclophosphamide (Sigma)**	750	83.20	20	4.16 (83.2)
**Doxorubicin (Sigma)**	50	5.55	2	2.75 (5.5)
**Vincristine (Sigma)**	1.4	0.16	1	0.16 (0.16)
**Prednisolone (Sigma)**	100	11.09	20	0.55 (11.1)
**Total Dose**	901.4	100	10	made up to 10

Note: The final concentration of CHOP is 10 mg/ml. The aliquots were stored at −80°C.
* indicates the concentration of each drug prepared in DMSO.

Supplementary Table S3: List of primary antibodies

Name of antibody	Type	Company	Cat No	Application	Dilution
**β-actin**	Mouse	Sigma	A5316	WB	1:10,000
**BSA**	Mouse	Sigma	B2901	WB	1:5000
**Cathepsin D**	Rabbit	Abcam	ab72915	WB	1:1000
**Cathepsin D**	Mouse	Sigma	C0715	IF	1:100
**CD80-PE**	Mouse	Biolegend	305208	FC	1:20
**CD83-PE**	Mouse	Biolegend	305308	FC	1:20
**CRT-PE**	Mouse	Enzo	ADI-SPA-601PE	FC	5 μg/ml
**CRT**	Rabbit	Cell Signalling	12238	WB	1:1000
**GA-101**	Human	Roche		Treatment	10 μg/ml
**eIF2α**	Rabbit	Cell Signalling	9722	WB	1:1000
**eIF2α-P^{S51}**	Rabbit	Cell Signalling	9721	WB	1:1000
**HMGB1**	Rabbit	Abcam	ab18256	IP	1:100
				IF	1:200
**HMGB1**	Mouse	Sigma	WH0003146M8	WB	1:1000
				IF	1:200
**OKT3**	Mouse	Abcam	ab86883	Treatment	10 μg/ml
**Rituximab**	Chimeric	Roche		Treatment	10 μg/ml
**S6-ribosome protein**	Rabbit	Cell Signalling	2217	IF	1:100
**STAT3**	Mouse	Cell Signalling	9139	WB	1:1000
**STAT3-P^{S727}**	Rabbit	ImmunoWay	YP0250	WB	1:1000
				IP	1:100
**STAT3-P^{S727}**	Rabbit	Cell Signalling	9134	IF	1:100
**STAT3-P^{Y705}**	Mouse	Cell Signalling	9138	WB	1:1000
				IP	1:100
**STAT3-P^{Y705}**	Rabbit	Cell Signalling	9145	WB	1:1000
				IP	1:100
				IF	1:100
**PARP**	Rabbit	Beyotime Biotec	AP102	WB	1:1000
**β-tubulin**	Mouse	Abmart	M 20005	WB	1:1000

Note: FC = flow cytometry; IF = Immuno-fluorescent staining; IP = Immuno-precipitation; WB = Western blotting.

Supplementary Table S4: List of secondary antibodies

Name of antibody	Type	Company	Cat No.	Application	Dilution
Anti-mouse IgG -HRP	Goat	Santa Cruz	sc-2005	WB	1:5000
Anti-rabbit IgG -HRP	Goat	Santa Cruz	sc-2004	WB	1:5000
Alexa Fluor® 488 Anti-Rabbit IgG	Goat	Invitrogen	A11034	IF, FC	1:100
Alexa Fluor® 546 Anti-rabbit IgG	Goat	Invitrogen	A11035	IF, FC	1:100
Alexa Fluor® 488 Anti-Mouse IgG	Donkey	Invitrogen	A21202	IF, FC	1:100
Alexa Fluor® 546 Anti-Mouse IgG	Donkey	Invitrogen	A10036	IF, FC	1:100
